# Supplementary material for: Supervised Kohonen Self-Organizing Maps of Acute Asthma from Air Pollution Exposure
Source: Int J Environ Res Public Health. 2021 Oct 21;18(21):11071. doi: 10.3390/ijerph182111071 (PMC8582892; doi:10.3390/ijerph182111071)
Supplement: Supplementary file 1 [file ijerph-18-11071-s001.zip › ijerph-1384069sup fin.pdf]

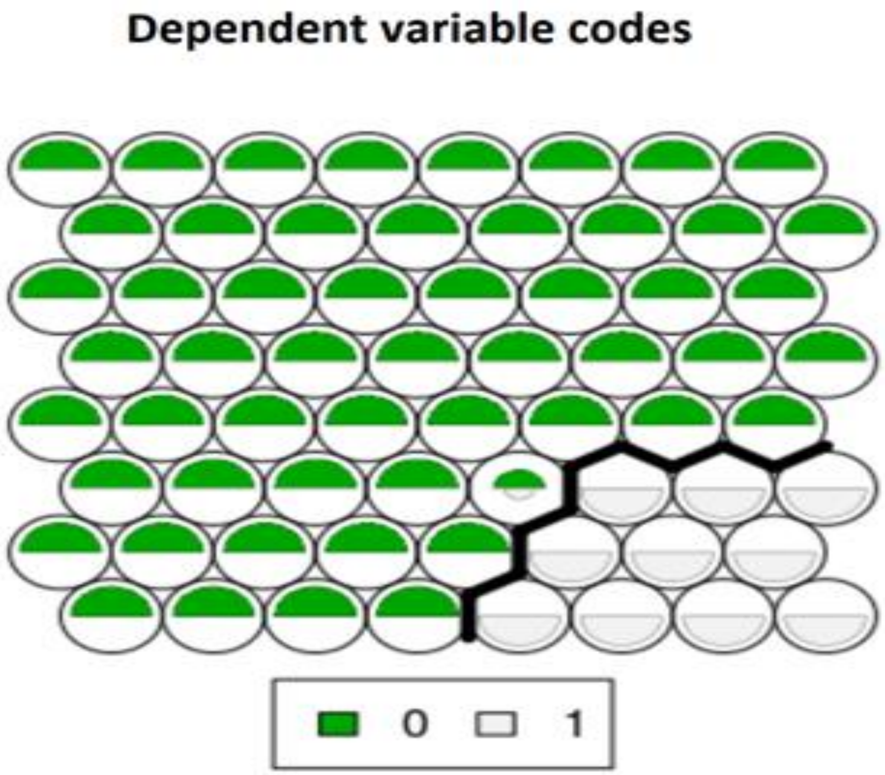

Figure S2 Supervised SOM code plots showing both dependent and independent variables.
